# Computational Analysis Suggests That AsnGTT 3′-tRNA-Derived Fragments Are Potential Biomarkers in Papillary Thyroid Carcinoma

**DOI:** 10.3390/ijms251910631

**Published:** 2024-10-02

**Authors:** Annie N. Do, Shruti Magesh, Matthew Uzelac, Tianyi Chen, Wei Tse Li, Michael Bouvet, Kevin T. Brumund, Jessica Wang-Rodriguez, Weg M. Ongkeko

**Affiliations:** 1 Research Service, VA San Diego Healthcare System, San Diego, CA 92161, USA; a9do@ucsd.edu (A.N.D.); smagesh@ucsd.edu (S.M.); muzelac@ucsd.edu (M.U.); tic015@ucsd.edu (T.C.); harrison.li@ucsf.edu (W.T.L.); 2 Department of Otolaryngology-Head and Neck Surgery, UC San Diego School of Medicine, University of California, La Jolla, CA 92093, USA; kbrumund@health.ucsd.edu; 3 Stanford School of Medicine, Stanford University, Stanford, CA 94305, USA; 4 UCSF School of Medicine, University of California, San Francisco, CA 94143, USA; 5 Department of Surgery, UC San Diego School of Medicine, University of Calfornia, La Jolla, CA 92093, USA; mbouvet@health.ucsd.edu; 6 Department of Surgery, VA San Diego Healthcare System, San Diego, CA 92161, USA; 7 Pathology Service, VA San Diego Healthcare System, San Diego, CA 92161, USA; jessica.wang-rodriguez@va.gov; 8 Department of Pathology, UC San Diego School of Medicine, University of California, La Jolla, CA 92093, USA

**Keywords:** tRNA-derived fragment, tRF, non-coding RNA, biomarker, THCA, PTC, thyroid cancer, thyroid carcinoma

## Abstract

Transfer-RNA-derived fragments (tRFs) are a novel class of small non-coding RNAs that have been implicated in oncogenesis. tRFs may act as post-transcriptional regulators by recruiting AGO proteins and binding to highly complementary regions of mRNA at seed regions, resulting in the knockdown of the transcript. Therefore, tRFs may be critical to tumorigenesis and warrant investigation as potential biomarkers. Meanwhile, the incidence of papillary thyroid carcinoma (PTC) has increased in recent decades and current diagnostic technology stands to benefit from new detection methods. Although small non-coding RNAs have been studied for their role in oncogenesis, there is currently no standard for their use as PTC biomarkers, and tRFs are especially underexplored. Accordingly, we aim to identify dysregulated tRFs in PTC that may serve as biomarker candidates. We identified dysregulated tRFs and driver genes between PTC primary tumor samples (n = 511) and adjacent normal tissue samples (n = 59). Expression data were obtained from MINTbase v2.0 and The Cancer Genome Atlas. Dysregulated tRFs and genes were analyzed in tandem to find pairs with anticorrelated expression. Significantly anticorrelated tRF-gene pairs were then tested for potential binding affinity using RNA22—if a heteroduplex can form via complementary binding, this would support the hypothesized RNA silencing mechanism. Four tRFs were significantly dysregulated in PTC tissue (*p* < 0.05), with only AsnGTT 3′-tRF being upregulated. Binding affinity analysis revealed that tRF-30-RY73W0K5KKOV (AsnGTT 3′-tRF) exhibits sufficient complementarity to potentially bind to and regulate transcripts of SLC26A4, SLC5A8, DIO2, and TPO, which were all found to be downregulated in PTC tissue. In the present study, we identified dysregulated tRFs in PTC and found that AsnGTT 3′-tRF is a potential post-transcriptional regulator and biomarker.

## 1. Introduction

The incidence rate for thyroid cancer has more than tripled in the United States from 1975 to 2013 [1], ranking ninth out of 36 cancers in a global survey conducted in 2020 [2]. Although they are generally considered to have better prognosis than other cancers, papillary thyroid carcinomas (PTCs) account for over 80% of thyroid cancer diagnoses and are the primary cause for increasing incidence [1]. Previous research has suggested that this trend is driven by overdiagnosis, in which patients with low-risk and indolent thyroid cancers are unnecessarily diagnosed and treated. To remedy this issue, professional guidelines for diagnosing and classifying PTC have been altered to be more conservative. Some of these changes include raising size thresholds for thyroid nodules and narrowing the histological framework for thyroid carcinomas [3].

Currently, imaging and cytological evaluation via fine needle aspiration are the two standard methods for diagnosing PTC. However, they remain limited in their ability to accurately detect thyroid carcinomas of ambiguous presentation, posing challenges for clinicians evaluating whether patients should receive surgical intervention or follow-ups. Consequently, efforts have been made to develop new strategies for detecting and evaluating PTC, particularly on the front of molecular markers [4]. Studies have shown that, when used in conjunction with imaging and cytology, genetic markers such as the BRAFV600E mutation can improve diagnostic accuracy for PTC [5,6]. However, limited efforts have been made to identify biomarkers from the non-coding RNA genome. While certain long non-coding RNAs, such as HOTAIR and MALAT1, have been identified as potential biomarkers in various studies, far less evidence exists for the use of small non-coding RNAs as biomarkers [4].

One class of non-coding RNA that has been poorly studied is transfer RNA-derived fragments (tRFs), a class of small non-coding RNA that is derived from pre-tRNA and mature tRNA [7,8]. Generally, tRFs are classified and named by the tRNA from which they were derived, including both the amino acid and the anti-codon of the tRNA. tRFs are defined as either 3′, 5′, internal, or half, depending on their location on the tRNA before cleavage. They have been found in both the nucleus and cytoplasm, with 3′-tRFs being more common to the cytoplasm and 5′-tRFs being more common to the nucleus [9]. Several tRFs have been found to be significantly dysregulated in human cancers [10], and in some cases, have been found to be mechanistically involved in tumorigenesis through the regulation of gene expression [11,12,13,14].

More specifically, tRFs have been found to modulate gene expression through the formation of an RNA-induced silencing complex (RISC). The RISC mechanism is currently one of the most well-documented hypotheses for tRF involvement in gene regulation and provides the theoretical framework for this study. It has been shown that tRFs associate with Argonaute (AGO) proteins that are capable of destabilizing and downregulating mRNA upon the binding of the complex and transcript [9,15,16,17,18]. Binding of the RISC and transcript occurs when the tRF involved in the RISC exhibits highly specific base-pair complementarity to the mRNA. Sites involved in binding are referred to as seed regions, and typically span less than 10 nucleotides [9,15]. This mechanism results in the silencing of the transcript by the RISC and can, thus, alter the expression of genes that are critical to cell survival and proliferation. tRFs involved in RISCs are, therefore, biologically relevant to oncogenesis and may serve as prognostic or diagnostic biomarkers in cancer. Accordingly, tRFs derived from both tissue and liquid biopsy have been proposed as biomarkers in several cancers—including breast cancer, lung adenocarcinoma, gastric cancer, colorectal cancer, and head and neck squamous cell carcinoma [19,20,21,22,23,24]. However, due to the relative recency of the discovery of tRFs, further investigation into their biological properties and functional mechanisms is required to determine their validity as biomarkers in human diseases.

Despite growing evidence for the relevance of tRFs to oncogenesis and their potential as biomarkers, the role of tRFs in PTC has yet to be robustly explored. As such, this study aims to examine the relationship between tRNA-derived fragments (tRFs) and known PTC driver genes. To do so, we obtained a dataset of tRF expression from MINTbase v2.0 for 511 primary PTC tumor samples and 59 adjacent normal tissue samples. All patient samples are derived from The Cancer Genome Atlas, from which we also obtained patients’ gene expression values. We identified 4 tRFs and seven genes that were dysregulated in PTC tissue through differential expression analysis. Next, we calculated Spearman’s rank correlation coefficient for pairs of dysregulated tRFs and dysregulated genes. Significantly anticorrelated (*p* < 0.05, r < 0) tRF-gene pairs were then tested for binding affinity using RNA22; if greater expression of the tRF correlates to lower gene expression, and the tRF and mRNA can form a heteroduplex via complementary binding, this would support the hypothesized RNA interruption mechanism. Ultimately, we identified 4 tRF-gene pairs that displayed significantly anticorrelated expression and potential binding affinity through simulation, suggesting the possibility of a RISC involving the tRF and target transcript. These findings can guide future inquiries into the use of tRFs as diagnostic or prognostic biomarkers in PTC and contribute to the growing body of evidence on the relevance of non-coding RNA to cancer.

## 2. Results

### 2.1. Differentially Expressed tRFs and PTC Driver Genes

**Figure 1:**
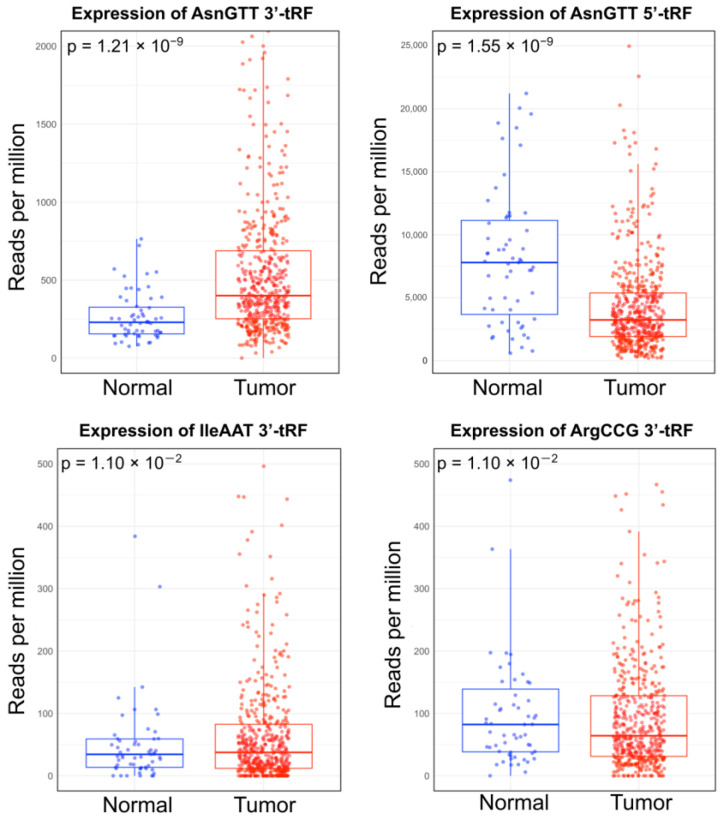
Box plots depicting the differential expression of tRFs between primary papillary thyroid carcinoma (PTC), primary tumor samples (n = 511), and normal samples (n = 59). All results were filtered by *p* < 0.05 following the Benjamini–Hochberg correction.

Differential expression analysis on the tRF expression of PTC primary tumor samples (n = 511) and normal tissue samples (n = 59) revealed statistically significant differences in the tRF landscape of cancer samples (Figure 1). All *p*-values were adjusted using the Benjamini–Hochberg correction method and filtered for *p* < 0.05 (Table 1).

AsnGTT 3′-tRF was found to be significantly upregulated in cancer samples (log_2_FC = 1.20 × 10^0^), while AsnGTT 5′-tRF, ArgCCG 3′-tRF, and IleAAT 3′-tRF were found to be significantly downregulated in cancer samples (log_2_FC of −9.41 × 10^−1^, −7.91 × 10^−1^, and −1.00 × 10^0^, respectively). AsnGTT 3′-tRF also had the lowest *p*-value after adjustment (*p* = 1.21 × 10^−9^), with AsnGTT 5′-tRF having the second lowest (*p* = 1.55 × 10^−9^). Comparatively, ArgCCG 3′-tRF and IleAAT 3′-tRF had higher, identical *p*-values after adjustment (*p* = 1.10 × 10^−2^).

**Figure 2:**
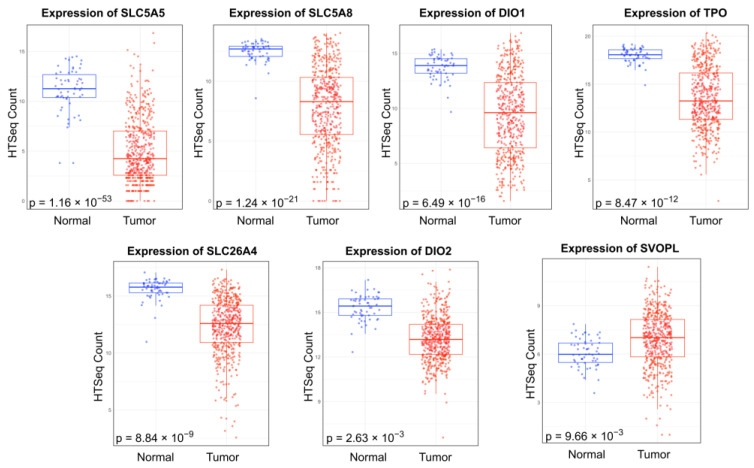
Box plots depicting the differential expression of genes that have been implicated in thyroid cancer between primary papillary thyroid carcinoma (PTC) primary tumor samples (n = 511) and normal samples (n = 59). All results were filtered by *p* < 0.05 following the Benjamini–Hochberg correction.

Similarly, differential expression of 22 THCA driver genes was analyzed between PTC and normal tissue samples, revealing seven dysregulated genes: SLC5A5, SLC5A8, SLC26A4, DIO1, DIO2, TPO, and SVOPL (Figure 2). All *p*-values were adjusted using the Benjamini–Hochberg correction method and filtered for *p* < 0.05. SVOPL was the only gene found to be upregulated in cancer samples; all other dysregulated genes were downregulated in PTC tissue samples (Table 1).

### 2.2. Negative Correlation between the Expression of Dysregulated tRFs and Dysregulated Genes

**Figure 3:**
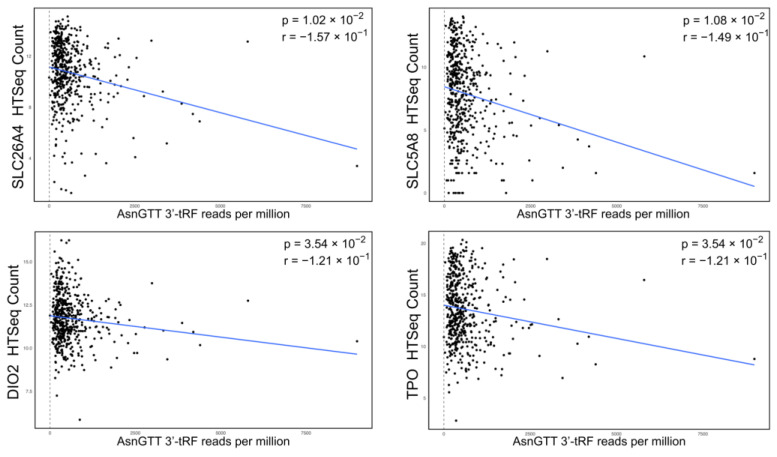
Scatterplots showing the negative relationship between tRF expression and gene expression (r < 0, *p* < 0.05). Gene expression was plotted against tRF expression and formatted with trendlines. All results were filtered by *p* < 0.05 following the Benjamini–Hochberg correction.

To characterize the relationship between the expression of dysregulated tRFs and dysregulated genes, Spearman’s rank correlation coefficient was calculated. In accordance with the hypothesis that a tRF-assisted RISC can knockdown mRNA transcripts, pairings were selected for significant anticorrelated expression (r < 0, *p* < 0.05), where greater tRF expression was associated with lower gene expression. All *p*-values were adjusted using the Benjamini–Hochberg correction method. We found four pairs consistent with this trend (Figure 3). Notably, all pairs involved AsnGTT 3′-tRF, an upregulated tRF, with downregulated genes (SLC26A4, SLC5A8, TPO, and DIO2).

### 2.3. Binding Affinity Analysis

It is hypothesized that tRFs bind to mRNA at complementary sites along the transcript during the formation of a RISC. The sites along the tRF involved in binding are referred to as seed regions and are typically less than 10 base pairs in length. Accordingly, the four anticorrelated tRF-gene pairs were tested for potential binding affinity by identifying seed regions with the software RNA22. All tRF sequences classified as an AsnGTT 3′-tRF on MINTbase v2.0 were included in this analysis to account for variability in sequences; for clarity, unique tRF sequences are referred to by their identifying license plate consistent with MINTbase v2.0 nomenclature.

**Figure 4:**
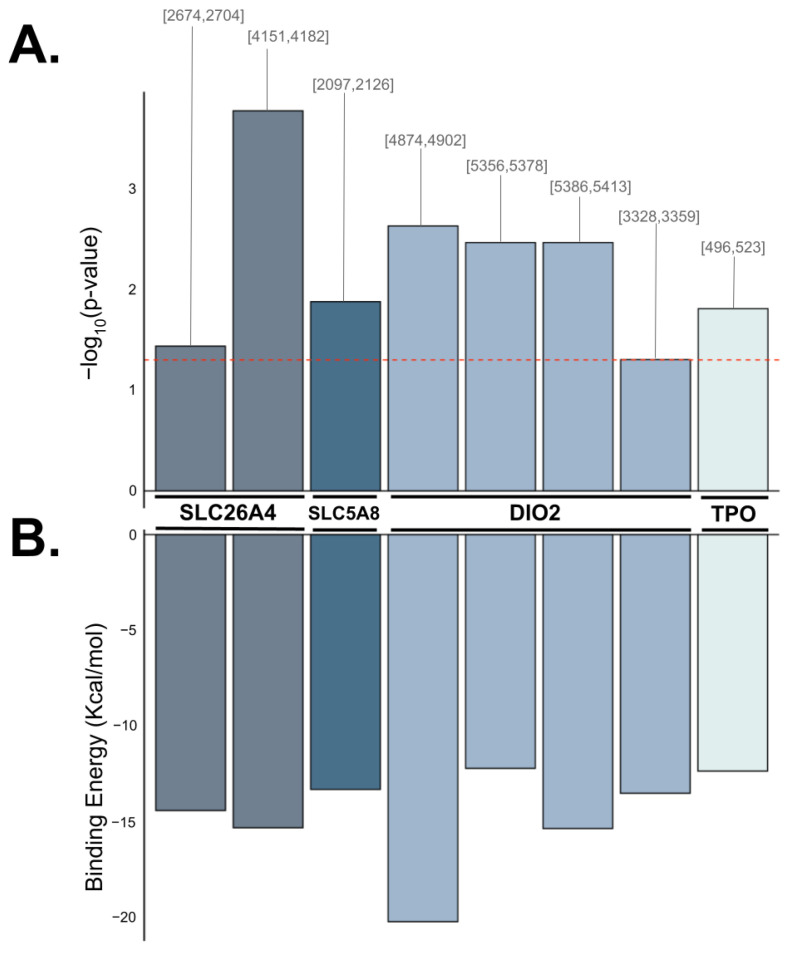
Binding affinity between anticorrelated tRF-gene pairs was predicted using base pair complementarity. Bar plots depicting each instance of predicted binding between tRF-30-RY73W0K5KKOV (AsnGTT 3′-tRF) and the target gene (SLC26A4, SLC5A8, DIO2, and TPO). (**A**) shows the negative logarithm of the test statistic and the target region’s start and end position in brackets. The dotted red line indicates the benchmark for statistical significance (−log(0.05)). (**B**) depicts the corresponding binding energy predicted to be released by the heteroduplex in Kcal/mol.

All anticorrelated pairs were found to exhibit sufficient complementarity for binding (*p* < 0.05) and involved one specific AsnGTT 3′-tRF (tRF-30-RY73W0K5KKOV) in common. The anticorrelated tRF-gene pairs with predicted binding capability consist of AsnGTT 3′-tRF and SLC26A4, AsnGTT 3′-tRF and SLC5A8, AsnGTT 3′-tRF and DIO2, and AsnGTT 3′-tRF and TPO (Figure 4). These tRFs and genes are predicted to contain sufficient complementarity to recruit a silencing complex, leading to the degradation of these mRNA transcripts. tRF-30-RY73W0K5KKOV is predicted to bind to DIO2 at three target sites and to SLC26A4 at two target sites. tRF-30-RY73W0K5KKOV is also predicted to bind to SLC5A8 and TPO at a single target site. Additionally, tRF-23-7SFR06RDDV (also an AsnGTT 3′-tRF) was predicted to bind to DIO2, but not to any other gene (Table 2).

## 3. Discussion

The utilization of non-coding RNAs as biomarkers for cancer has garnered significant attention in recent years, particularly focusing on the small non-coding RNA category. These small non-coding RNA, typically less than 200 nucleotides in length, include various subcategories such as microRNAs, transfer RNAs, and endogenous small interfering RNAs [25]. Among these, microRNAs have been studied most extensively, with many being characterized as “onco-miRs”. These molecules have been detected in the cell-free component of blood and have shown potential as biomarkers in multiple cancer types due to their stability and detectability [25,26]. tRFs are another classification of small non-coding RNA, yet their role in tumorigenesis has not been as widely studied. Given the functional diversity of small non-coding RNA and the statistically significant correlation between tRF abundance and gene expression demonstrated by our analysis, there is a strong rationale for exploring tRFs as potential cancer biomarkers.

A commonly proposed mechanism of tRFs involves the formation of RNA-induced silencing complexes (RISC) with Argonaute (AGO) proteins, which, in turn, bind to mRNA and result in enzymatic knockdown of the transcript [9,15,16,17,18]. The exact mechanism by which mRNA silencing is accomplished depends on the AGO protein involved. Currently, only AGO2 has been consistently shown to possess direct mRNA-cleaving abilities. However, there is evidence indicating that AGO1 and AGO3 are also involved in post-transcriptional regulation, with AGO3 being capable of slicing mRNA under more limited conditions than AGO2 [15,27]. A potential difference between the tRF-directed mechanism and that of other non-coding RNA is the specific AGO protein involved in the RISC. For instance, microRNAs are known to associate with the AGO2 proteins, which have been primarily identified in the nucleus [28]. Contrastingly, studies have observed that tRFs associate with AGO1, AGO3, and AGO4 [9,17]. This is especially relevant for 3′-tRFs, as this class of tRF is primarily found in the cytoplasm [9]. Consequently, dysregulation in tRF abundance may result in changes in the expression of certain genes through association with AGO proteins.

We identified four tRFs that are dysregulated in PTC primary tumor tissue, with AsnGTT 3′-tRF being the most significantly dysregulated tRF (adjusted *p*-value = 1.21 × 10^−9^) and the only tRF that was upregulated (log_2_FC = 1.20 × 10^0^). As previously discussed, 3′-tRFs are primarily cytoplasmic, making plausible their association with AGO1, AGO3, and AGO4 proteins. Moreover, AsnGTT 3′-tRF was found to be significantly upregulated by Shan et al. in an independent study that conducted high-throughput next-generation sequencing on four PTC patient cancer samples and adjacent normal tissue samples [29]. The findings in this study expand upon Shan et al.’s by exploring the correlation between AsnGTT 3′-tRF and PTC driver genes in silico, while also corroborating their differential expression analysis. However, this study focuses on the AsnGTT 3′-tRFs tRF-30-RY73W0K5KKOV and tRF-23-7SFR06RDDV, while Shan et al. identified tRF-17-K5KKOV2. This variation suggests that several tRFs within the AsnGTT 3′-tRF class may be implicated in PTC development. It also highlights the importance of maintaining distinctions between tRFs within a class, as the precise cellular function of a tRF may depend on its unique structure and ability to interact with other molecules. Although this study did not predict tRF-gene interactions involving tRF-17-K5KKOV2, future studies may explore seed regions between tRF-17-K5KKOV2 and other molecular targets. Overall, the convergence of our findings and Shan et al.’s suggests that AsnGTT 3′-tRF may play a critical role in thyroid cancer development and supports the hypothesis that it may serve as a biomarker for PTC.

Contrastingly, we found AsnGTT 5′-tRF (adjusted *p*-value = 1.55 × 10^−9^), IleAAT 3′-tRF (adjusted *p*-value = 1.10 × 10^−2^) and ArgCCG 3′-tRF (adjusted *p*-value = 1.10 × 10^−2^) to be significantly downregulated in cancer samples compared to normal tissues. However, to our knowledge, the dysregulation of these three tRFs in PTC has not yet been reported in the previous literature. Notably, a limitation of this study is its relatively small normal sample size; The Cancer Genome Atlas contained 59 adjacent normal tissue samples. Consequently, the proportion of normal samples to tumor samples (n = 511) is low, potentially impacting the statistical accuracy of the differential expression analysis and subsequent conclusions. Thus, future studies should seek to validate the expressional dysregulation of these tRFs in PTC tissue. Additionally, this study’s binding affinity analysis did not reveal potential tRF-gene interactions involving these downregulated tRFs. Future studies may, therefore, investigate the molecular targets of these tRFs in order to explore the possibility of using AsnGTT 5′-tRF, IleAAT 3′-tRF, and ArgCCG 3′-tRF as biomarkers in PTC.

Among the 22 genes associated with thyroid pathology analyzed in this study, seven were identified as significantly dysregulated in their expression, with all but one gene (SVOPL) being downregulated in cancer samples. The gene SLC5A5 exhibited the most negative log_2_FC of −1.08 × 10^0^ (adjusted *p*-value = 1.16 × 10^−53^). This gene encodes the sodium iodide symporter, a highly specialized transmembrane glycoprotein that plays a significant role in transport of iodide across the basolateral membrane of follicular cells [30]. The sodium iodide symporter is one of several key proteins that participate in the thyroid’s processing of iodide; other proteins include pendrin, thyroid peroxidase, and thyroglobulin. In thyroid carcinomas, downregulation of SLC5A5 is correlated to poor differentiation, locoregional recurrence, and distant metastases [31,32]. As such, downregulation of SLC5A5 may disrupt essential thyroid function and contribute to oncogenesis.

Another member of the solute carrier family, SLC5A8, was downregulated in cancer samples (log_2_FC = −6.09 × 10^−1^; adjusted *p*-value = 1.24 × 10^−21^). SLC5A8 encodes a sodium-coupled monocarboxylate transporter protein and has been noted by various studies to function as a tumor suppressor [33,34]. As a tumor suppressor, SLC5A8 has been observed to induce apoptosis by transporting short-chain fatty acids into tumor cells [35]. The short-chain fatty acids may then inhibit histone deacetylases, allowing the cell to express genes that facilitate cell death. One study found that SLC5A8 expression is significantly reduced in PTC, with a 40-fold downregulation associated with hypermethylation at exon one [36]. Accordingly, downregulation of this tumor suppressor may promote tumorigenesis by allowing cells to evade apoptosis.

Furthermore, SLC26A4, which was also downregulated in PTC (log_2_FC = −3.31× 10^−1^; adjusted *p*-value = 8.84 × 10^−9^), has been implicated in thyroid cancer pathogenesis. SLC26A4 encodes pendrin, an anion-exchanging glycoprotein that facilitates the efflux of iodide into the follicular lumen, where it can be used for hormone synthesis. While pendrin’s role in iodide transport is not as salient as that of the sodium iodide symporter (SLC5A5), its expression is still believed to be important for thyroid function and health [37]. Higher expression of pendrin has been found to correlate to greater differentiation in thyroid carcinoma, while hypermethylation of pendrin has been found to be a critical event in early tumorigenesis [38,39]. Additionally, it has been suggested that lower levels of pendrin allow tumor cells to maintain an elevated pH through the inhibition of anion exchange [40]. As such, downregulation of SLC26A4 may promote tumorigenesis.

The DIO2 gene, which encodes type 2 deiodinase, is crucial for thyroid hormone activation. Specifically, DIO2 is known to convert the pro-hormone thyroxine into triiodothyronine, which is then supplied to the nucleus. For the last two decades, DIO2 has been consistently found to be downregulated in PTC compared to normal tissue. This is also supported by our findings (log_2_FC = −1.85 × 10^−1^; adjusted *p*-value = 2.63 × 10^−3^). The precise molecular mechanism implicating DIO2 and triiodothyronine in PTC remains unknown; however, the current literature supports that decreased levels of intracellular thyroid hormone are important to PTC tumorigenesis [41].

Finally, the TPO gene was downregulated with log_2_FC = −3.56 × 10^−1^ and adjusted *p*-value = 8.47 × 10^−12^. TPO encodes thyroperoxidase, which has a crucial role in incorporating iodine into thyroglobulin to synthesize thyroid hormones [42]. Its downregulation could disrupt this process, resulting in hormonal imbalance and loss of thyroid function. Notably, a prior study validated the association between TPO expression levels and lymph node metastasis in patients with PTC. Functional enrichment analysis indicated that TPO is significantly linked to pathways involved in amino acid metabolism, gene expression regulation, and tumorigenesis [43]. Additionally, reduced TPO expression was found to correlate with increased metastatic recurrence in PTC.

We next investigated the relationship between the dysregulation of tRFs and specific genes by identifying pairs that exhibit an anti-correlational relationship. According to the proposed tRF and AGO-mediated knockdown of genes, increased expression of a particular tRF is expected to correspond to lower expression of the target gene. In other words, the tRF and gene expression should demonstrate an anti-correlated relationship. Furthermore, the RISC associates with the transcript through complementary binding between the tRF and transcript at seed regions, leading to gene knockdown via association with AGO proteins. Therefore, we followed correlational analysis with binding affinity analysis to confirm whether the proposed mechanism is substantiated by the identification of seed regions between the sequence of the tRFs and genes’ coding cDNA transcripts.

Spearman’s rank correlation coefficient analysis of dysregulated tRF and dysregulated gene expression levels revealed four tRF-gene pairs that are anti-correlated. All pairs exhibited modest correlational strength, with the most notable being AsnGTT 3′-tRF and SLC26A4 (r = −0.157; adjusted *p*-value = 1.02 × 10^−2^). Additional tRF-gene pairs with anti-correlational relationship include AsnGTT 3′-tRF and SLC5A8, AsnGTT 3′-tRF and DIO2, and AsnGTT 3′-tRF and TPO. Notably, all four pairs involve AsnGTT 3′-tRF, which we found to be upregulated in tumor samples, and genes that we found to be downregulated in tumors. The opposing direction of dysregulation is consistent with the proposed AGO-associated mechanism; the upregulation of a tRF that targets a certain transcript may result in the formation of a RISC that contributes to the downregulation of the gene.

The results from binding affinity analysis further supports this hypothesis, as all four tRF-gene pairs yielded at least one possible seed region for binding. The goal of this analysis was to test whether the anti-correlated tRF-gene pairs exhibit sufficient base-pair complementarity to form seed regions. If a potential seed region exists, the anti-correlation between the expressions of the dysregulated gene and dysregulated tRF may be plausibly explained by the AGO-associated RNA silencing mechanism. Remarkably, while 57 unique tRF sequences have been classified as AsnGTT 3′-tRF on MINTbase v2.0, seven of the eight seed regions identified in our analysis involved the tRF-30-RY73W0K5KKOV sequence. In particular, tRF-30-RY73W0K5KKOV exhibits three potential seed regions with DIO2, suggesting that this target gene may be an especially likely candidate for tRF-mediated regulation.

It is important to note that this study is limited by being computational in nature and requires in vitro and in vivo investigation to validate its results. Although this study proposes tRF-gene pairs that may be involved in the progression of PTC, more evidence must be provided to validate the hypothesized mechanism of tRF-mediated gene regulation. Likewise, in vitro and in vivo experimentation is required to validate the potential for AsnGTT 3′-tRF to act as a biomarker in PTC. Specifically, to advance the application of tRF-30-RY73W0K5KKOV in clinical diagnostics, future studies should observe its functional behavior within the cell and explore its binding affinity with the aforementioned target genes. Studies should also investigate whether tRF-30-RY73W0K5KKOV, or any other form of AsnGTT 3′-tRF, associates with AGO proteins in thyroid cells. Accordingly, studies could observe the downstream mechanistic effects of gene regulation and evaluate the influence of tRF-gene interactions on PTC development.

If the functional significance of AsnGTT 3′-tRF in PTC is validated, it may also be investigated as a potential therapeutic target. Inhibiting techniques may be developed to lower the expression of this tRF, with the goal of restoring expression levels of the target gene(s). A study by Lu et al. demonstrates the clinical potential for such techniques using in vitro PTC cell lines and in vivo mouse models; an upregulated SerAGA 3′-tRF (tRF-18-H7PU4HD2) was found to target KIF1B, hinder apoptosis, and enhance cell proliferation in PTC [44]. Lu et al. found that PTC tumors with inhibited tRF expression were significantly smaller than those without. Similarly, a study by Han et al. found that an upregulated 5′-tRNA half (tiRNA-Gly) promotes alternative splicing and oncogenesis in PTC; Han et al. also found that knockdown of tiRNA-Gly reduced tumor sizes in mouse models, strengthening the rationale for investigating tRFs in PTC [45]. Accordingly, the precise molecular mechanisms and pathways involving AsnGTT 3′-tRF should be elucidated using in vitro and in vivo experimentation so that a foundation for therapeutic exploration may be established.

## 4. Materials and Methods

### 4.1. Data Acquisition

All tRF expression values were extracted from MINTbase v2.0 and can be found using the query “thyroid carcinoma”. A total of 511 primary tumor samples and 59 normal samples were obtained by downloading a zip file with patient tRF expression profiles (https://cm.jefferson.edu/tcga-mintmap-profiles/ accessed on 17 May 2023). Sample IDs correspond to samples of the THCA cohort on The Cancer Genome Atlas (TCGA). Gene expression data for the THCA cohort were obtained from the GDC repository (https://portal.gdc.cancer.gov/projects/TCGA-THCA accessed on 17 May 2023).

Female patients comprise 72.56% of samples while male patients comprise 26.64% of samples. Patients aged 39 and younger comprise 35.79% of samples, patients between ages 40 and 59 comprise 39.76% of samples, and patients aged 60 and older comprise 23.66% of samples. No sex or age data was available for 0.80% of samples.

Multiple races are represented in the sample set. Patients identified as white comprise 65.81% of samples; patients identified as Asian comprise 10.14% of samples; patients identified as black or African American comprise 5.37% of samples; patients identified as American Indian or Alaska native comprised 0.20% of samples. No racial data were available for 18.48% of samples.

Within the sample set, 55.86% of tumors were diagnosed as pathologic stage I; 10.14% of tumors were diagnosed as pathologic stage II; 21.87% of tumors were diagnosed as pathologic stage III; 9.34% of tumors were diagnosed as pathologic stage IVA; 1.19% of tumors were diagnosed as pathologic stage IVC. No pathologic staging data were available for 1.60% of tumor samples.

### 4.2. Differential Expression Analysis

Differential expression analysis was conducted with edgeR library for tRF abundance and gene expression of cancer and normal samples. Significance was identified and filtered by FDR < 0.05 after adjusting the test statistic with the Benjamini–Hochberg correction method.

### 4.3. Anticorrelation of tRF Expression and Gene Expression

Spearman’s rank correlation coefficient was calculated in R to measure the strength and direction of correlation between dysregulated tRF expression and dysregulated gene expression. Significant results were identified and filtered by FDR < 0.05 and r < 0 after adjusting the test statistic with the Benjamini–Hochberg correction.

### 4.4. Binding Affinity

The software RNA22v2 was used to compute the binding affinities of tRF-gene pairs (default parameters were selected: 63% sensitivity, 61% specificity, seed size of 7 with maximum of 1 UN-paired base in seed, minimum of 12 paired bases in heteroduplex, maximum –12 Kcal/mol folding energy for heteroduplex, no limit to maximum G:U wobbles allowed in seed region). tRF sequences were obtained from MINTbase v2.0 by selecting for all tRFs classified under a certain amino acid, anticodon, and type (e.g., query AsnGTT 3′-tRF yielded 57 unique tRF sequences). Canonical cDNA gene sequences were obtained from the Ensembl database.

### 4.5. Plots

The R package ggplot2 was used to generate figures.

### 4.6. Lead Contact

Further information and requests for resources and reagents should be directed to and will be fulfilled by the lead contact, Weg M. Ongkeko (rongkeko@health.ucsd.edu).

## 5. Conclusions

We identified AsnGTT 3′-tRF as a significantly upregulated tRF in papillary thyroid carcinoma (PTC) primary tumor tissue. Subsequent analysis of AsnGTT 3′-tRF with downregulated PTC driver genes (SLC26A4, SLC5A8, DIO2, and TPO) revealed that the expression of these genes and AsnGTT 3′-tRF are significantly anticorrelated. Moreover, simulated binding affinity analysis predicts that tRF-30-RY73W0K5KKOV (a sequence of AsnGTT 3′-tRF) exhibits sufficient complementarity to bind to all 4 gene transcripts. These findings are consistent with the proposed mechanism of tRF post-transcriptional regulation by mRNA degradation and highlight the potential of AsnGTT 3′-tRF to serve as a prognostic and diagnostic marker in PTC. Overall, this study contributes to the growing body of evidence on the relevance of tRFs and non-coding RNA in cancer. These findings may guide future in vitro and in vivo studies validating the potential for AsnGTT 3′-tRF to serve as a biomarker or therapeutic target in PTC.

## Figures and Tables

**Table 1:** Differentially expressed tRFs and PTC driver genes.

Name	Expression Type	log_2_FC	Adjusted *p*-Value
**AsnGTT 3′-tRF**	tRNA-derived fragment	1.20 × 10^0^	1.21 × 10^−9^
**AsnGTT 5′-tRF**	tRNA-derived fragment	−9.41 × 10^−1^	1.55 × 10^−9^
**ArgCCG 3′-tRF**	tRNA-derived fragment	−7.91 × 10^−1^	1.10 × 10^−2^
**IleAAT 3′-tRF**	tRNA-derived fragment	−1.00 × 10^0^	1.10 × 10^−2^
**SLC5A5**	Gene	−1.08 × 10^0^	1.16 × 10^−53^
**SLC5A8**	Gene	−6.09 × 10^−1^	1.24 × 10^−21^
**DIO1**	Gene	−4.89 × 10^−1^	6.49 × 10^−16^
**TPO**	Gene	−3.56 × 10^−1^	8.47 × 10^−12^
**SLC26A4**	Gene	−3.31 × 10^−1^	8.84 × 10^−9^
**DIO2**	Gene	−1.85 × 10^−1^	2.63 × 10^−3^
**SVOPL**	Gene	2.31 × 10^−1^	9.66 × 10^−3^

**Table 2:** Predicted binding ability of AsnGTT 3′-tRFs and dysregulated genes.

tRF License Plate	Gene	Start/End of Target Site	-Kcal/mol	Target Site Sequence	tRF Sequence	Base Pairs Involved in Heteroduplex		*p*-Value
tRF-30-RY73W0K5KKOV	SLC26A4	[2674,2704]	−14.4	TTCAGTTAGAGTGAGTGCTGACCCAACAGCC	GGTGGTGGTTCGAGCCCACCCAGGGACGC	...........((.(.(((((.(((.(((((	))))).))).)).))))))...........	3.65 × 10−2
tRF-30-RY73W0K5KKOV	SLC26A4	[4151,4182]	−15.3	GTGGTGGCTGGCGGGCGCCTGTAGTCCCAGCT	GGTGGTGGTTCGAGCCCACCCAGGGACGC	............((((...((.(..(((((((	)))))).)..))).))))............	1.68 × 10−4
tRF-30-RY73W0K5KKOV	SLC5A8	[2097,2126]	−13.3	GAAAAAGAAGCATGT TTTGAGCTATAAATC	GGTGGTGGTTCGAGCCCACCCAGGGACGC	...............((((((((((.((((	)))).))))))))))...............	1.32 × 10−2
tRF-30-RY73W0K5KKOV	DIO2	[4874,4902]	−20.2	GCTTCCAGAATGGGGC TTAAGTACCAATC	GGTGGTGGTTCGAGCCCACCCAGGGACGC	............((((((((.((((((((	)))))))).)).))))))............	2.34 × 10−3
tRF-23-7SFR06RDDV	DIO2	[5356,5378]	−12.2	GAAGTCACTATTTTG GCTCAAAC	GTTCGAGCCCACCCAGGGACGCC	...(((.((.....(((((.(((	))).))))).....)).)))...	3.42 × 10−3
tRF-30-RY73W0K5KKOV	DIO2	[5386,5413]	−15.34	TTCTCCCCCTCCCCTCAAAAAGCCAACA	GGTGGTGGTTCGAGCCCACCCAGGGACGC	...((((......(((.((..((((((.	.))))))..)).)))........))))...	3.42 × 10−3
tRF-30-RY73W0K5KKOV	DIO2	[3328,3359]	−13.5	TTTCTGGAAGAAAGGCT GTGAAGGGCCAATG	GGTGGTGGTTCGAGCCCACCCAGGGACGC	.............((((.((((...((((((.	.))))))..)))))))).............	4.97 × 10−2
tRF-30-RY73W0K5KKOV	TPO	[496,523]	−12.34	CTGCCCCCAAAATGCCCAAACACTTGCC	GGTGGTGGTTCGAGCCCACCCAGGGACGC	.(((((.......(((....((((.(((	))).))))....).)).......))).)).	1.55 × 10−2

## Data Availability

This paper analyzes existing, publicly available data from MINTbase v2.0 (https://cm.jefferson.edu/tcga-mintmap-profiles/ (accessed on 29 September 2024)) and The Cancer Genome Atlas (https://portal.gdc.cancer.gov/projects/TCGA-THCA (accessed on 29 September 2024) phs000178). Any additional information required to reanalyze the data reported in this paper is available from the lead contact upon request.
